# Capitalizing on Cancer Specific Replication: Oncolytic Viruses as a Versatile Platform for the Enhancement of Cancer Immunotherapy Strategies

**DOI:** 10.3390/biomedicines4030021

**Published:** 2016-08-24

**Authors:** Donald Bastin, Scott R. Walsh, Meena Al Saigh, Yonghong Wan

**Affiliations:** McMaster Immunology Research Centre, Department of Pathology and Molecular Medicine, McMaster University, Hamilton, ON L8N 3Z5, Canada; bastind@mcmaster.ca (D.B.); scott.walsh22@gmail.com (S.R.W.); alsaigm@mcmaster.ca (M.A.S.)

**Keywords:** oncolytic virotherapy, cancer immunotherapy, antitumor immunity

## Abstract

The past decade has seen considerable excitement in the use of biological therapies in treating neoplastic disease. In particular, cancer immunotherapy and oncolytic virotherapy have emerged as two frontrunners in this regard with the first FDA approvals for agents in both categories being obtained in the last 5 years. It is becoming increasingly apparent that these two approaches are not mutually exclusive and that much of the therapeutic benefit obtained from the use of oncolytic viruses (OVs) is in fact the result of their immunotherapeutic function. Indeed, OVs have been shown to recruit and activate an antitumor immune response and much of the current work in this field centers around increasing this activity through strategies such as engineering genes for immunomodulators into OV backbones. Because of their broad immunostimulatory functions, OVs can also be rationally combined with a variety of other immunotherapeutic approaches including cancer vaccination strategies, adoptive cell transfer and checkpoint blockade. Therefore, while they are important therapeutics in their own right, the true power of OVs may lie in their ability to enhance the effectiveness of a wide range of immunotherapies.

## 1. Introduction

The plethora of side effects associated with current standard of care treatments for cancer stem from their limited ability to consistently distinguish between healthy and diseased tissue. While the development of targeted therapies has sought to overcome this issue, these strategies often fall prey to either acquired or intrinsic resistance in tumors which are by nature heterogeneous and highly mutable [1]. Biological therapies represent new approaches to surmounting limitations of traditional treatments and have generated considerable enthusiasm in the past decade. For example, cancer immunotherapy which encompasses a variety of strategies designed to induce an antitumor immune response was recognized in 2013 as the “breakthrough of the year” by Science magazine [2]. With FDA approval for checkpoint blockers such as ipilimumab in 2011 and nivolumab in 2014, cancer immunotherapeutic strategies are poised to become critical components in the oncologists’ arsenal. In parallel, the field of oncolytic virotherapy, which employs cancer-targeted viruses to directly kill or generate an immune response against tumors [3] has evolved to give us its own FDA approved product. The year 2015 saw regulatory approval for the use of Imlygic (formerly talimogene lapharevec, T-VEC) which is a herpes virus engineered to express GM-CSF (granulocyte macrophage colony stimulating factor) in advanced melanoma [4,5].

While advances in the fields of cancer immunotherapy and oncolytic virotherapy have ushered in a new era of promise, these treatments are not successful for all patients. For example, in a clinical trial using Imlygic in patients with advanced melanoma the durable response rate was 16% compared to 2% when treated with GM-CSF alone [4]. Similarly, 3 year survival for patients with metastatic melanoma treated with ipilimumab can range from 20% to 26% [6]. Thus, while promising, these therapies require further development and new strategies to achieve their goal of having widespread curative potential.

It is becoming increasingly accepted that oncolytic viruses (OVs) function as immunotherapeutic agents [7] and thus there is considerable overlap between the fields of immunotherapy and oncolytic virotherapy. OVs release a variety of molecular signals in the context of the tumor which can serve to render an immunologically inert tumor into a highly immunoreactive tumor. This concept has been extensively reviewed elsewhere [8,9]. Owing to the breadth of immunostimulatory actions of OVs as well as the increasing number of OVs in preclinical development, these agents represent versatile platforms with which specific niches within the field of cancer immunotherapy can be fulfilled. In this way they can be used to complement other immunotherapies and compensate for potential limitations of these approaches. For the remainder of this review we intend to provide background on OVs and their immunotherapeutic activity and to summarize current efforts in their use as combinational agents to enhance other immunotherapeutic approaches.

## 2. Oncolytic Viruses: Cancer Specific Killers

The therapeutic benefit of viral infection in cancer therapy was first described over a century ago [10] and several case reports outlining positive correlations between temporary tumor remissions and viral disease were made while the field of virology was still in its’ infancy [11]. Thus the concept of an “oncolytic virus” as a cancer-specific lytic agent is not new. However our ability to manipulate these entities and rationally design them into cancer fighting tools has drastically improved since their original description [11].

The tropism of many viruses for cancerous tissue is not surprising given the highly proliferative nature and immune dysregulation associated with oncogenic transformation and tumor establishment [12]. Indeed, each of the hallmarks of cancer as summarized by Hanahan and Weinberg [13] can in their own right be viewed as facilitators of viral infection [12]. For instance, to achieve rapid proliferation, cancer cells must generate adequate quantities of nucleotides for DNA synthesis. These same building blocks can serve as raw materials for viral genome replication of oncolytic DNA viruses, such as HSV-1 [14] and vaccinia virus (VacV) [15,16] and obviate the need for the dNTP synthesizing accessory proteins. Furthermore, antiviral programs such as the interferon (IFN) pathway have a variety of antiproliferative and pro-apoptotic effects [17] which work against several hallmarks of cancer. To this end it has been demonstrated that a variety of human cancer cell lines display defects in IFN signalling [18]. While this provides these cells with a means of outcompeting their non-transformed counterparts it has also been shown to cripple their protection against viral infection [19]. Thus the distinguishing feature of cancer targeting by OVs when compared to other therapies may be their ability to target a cancerous state rather than a specific pathway per se. In fact, evidence is emerging that the tumor selectivity of OVs extends beyond direct targeting of cancer cells to include infection of corrupted components of the tumor microenvironment such as cancer associated fibroblasts [20].

While the natural propensity of OVs to infect and lyse cancer cells provides a useful backbone off which to build, efforts to enhance the cancer-selectivity of OVs through genetic engineering have enabled much of the recent progress in the field [11]. This specificity can be achieved through strategies such as deletion of genes which are redundant in a cancerous environment but crucial in the infection of healthy cells. This has the effect of restricting productive infection to neoplastic tissue. For example, removal of the VacV thymidine kinase has been used to confine this OV to the tumor where elevated levels of endogenous thymidine triphosphate compensates for such deletions [15,16]. Vesicular stomatitis virus (VSV) and Maraba virus, OVs from the *Rhabdoviridae* family, have been lauded as ideal oncolytic agents. This is partially due to their low pathogenicity and established immunity in human populations as well as the relative ease with which they can be genetically engineered [21]. The matrix (M) protein of wild type VSV was shown to prevent nuclear export of mRNA. During VSV infection, this serves to curtail the host innate antiviral response by preventing expression of type I IFN genes [18]. Deletion of specific residues in the M protein of these viruses (VSVΔ51 and Maraba MG1) cripples their defense mechanisms against type I IFN thereby allowing them to replicate in IFN-deficient cancer cells while rendering them severely attenuated in healthy cells [18,21].

## 3. Revisiting the Paradigm: The Immune Response Is Paramount

While early interest in the field of oncolytic virotherapy revolved around the cancer-specific lytic (direct killing) ability of these agents, the first wave of clinical trials were widely regarded as disappointing [22]. Adenoviruses were amongst the first genetically engineered viruses to enter the clinical arena and while demonstrably safe, objective benefit from their use as single agents was not observed [23]. However in a trial combining an E1B 55-kDa deleted adenovirus (ONYX-015) with cisplatin and 5-FU chemotherapy, significant improvement in objective responses was observed when compared to chemotherapy alone [23,24] justifying further excitement. Subsequently it was shown that the therapeutic benefit arising from adenoviral vectors is critically dependent on their ability to illicit an immune response as opposed to their direct oncolytic activity [25]. Such findings have been corroborated for a variety of other cancer-targeted viruses [26,27,28]. Therefore, the immune system which was initially seen as a barrier to effective viral delivery is now widely regarded as an ally in oncolytic virotherapy [29].

**Figure 1:**
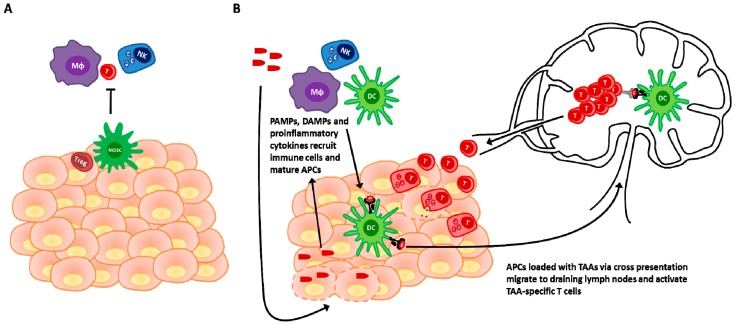
Immunostimulatory actions of oncolytic viruses converts non-inflamed tumors into inflamed tumors and induce an antitumor immune response. (**A**) Established tumors with a non-inflamed phenotype show a reduced inflammatory cytokine expression profile and a lack of T cell infiltration. A state of immune exclusion and ignorance is induced in these tumors by immunosuppression induced by inhibitory immune cells such as Treg and myeloid derived suppressor cells (MDSC) in the tumor microenvironment; (**B**) Infection of a tumor with an oncolytic virus leads to a variety of immunostimulatory actions which can convert a non-inflamed tumor into an inflamed tumor and promote an antitumor response. OV infection leads to the release of chemokines and cytokines from infected cells which recruit a variety of innate and adaptive immune effector cells. PAMPs (pathogen associated molecular patterns) associated with OVs and DAMPs (danger associated molecular patterns) released upon oncolysis provide maturation signals to antigen presenting cells within the tumor microenvironment which then phagocytose and cross-present these antigens in the secondary lymphoid organs to induce an adaptive anti-tumor response.

It is well documented that viral infection of a tissue stimulates the recruitment and activation of the immune system leading to elimination of infected cells and protection or immunopathology [30]. The immune activity of OVs can be viewed as paralleling this process in the context of a cancer restricted infection, activating tumor resident immune cells and recruiting additional effectors to the tumor microenvironment. In the past decade it has become widely appreciated that cancer becomes clinically apparent when the disease has acquired the capacity to evade immune detection or locally dampen the immune response [31]. As such, tumors can be separated into two immunophenotypes; non-inflamed and inflamed [32]. Non-inflamed tumors lack an appreciable sign of innate or adaptive immune activation and appear to resist immune attack through a mechanism of immune exclusions or ignorance. Inflamed tumors show a cytokine and chemokine profile consistent with innate immune activation as well as evidence of T cell infiltration into the tumor mass and appear to resist immune elimination through mechanisms of immune suppression and inhibition. This phenotype difference is associated with a clinical outcome difference where non-inflamed tumors are typically refractory to immunotherapies; and inflamed tumors showing a more favourable clinical outcome from immunotherapies [32]. A major strength of OV therapy is that OV infection of tumor tissue can convert a non-inflamed tumor (Figure 1A) into an inflamed tumor through the release of immunostimulatory mediators and the recruitment of immune cells (seen in Figure 1B and discussed in the following sections). Thereby, the responsiveness to immune attack and the clinical outcome achieved is enhanced in these tumors by OV mediated conversion of their inflammatory status.

### 3.1. Viral Detection and Immunogenic Cell Death in Innate Activation and Adaptive Priming

Immune activation in the context of tissue infection can be viewed as occurring through two complementary mechanisms, pathogen associated molecular patterns (PAMPs) and danger associated molecular patterns (DAMPs). PAMPs represent broadly expressed constituents of different classes of microbes which can be detected by invariant pattern recognition receptors (PRRs) on innate immune cells. This allows the immune system to recognize “infectious non-self” [33,34]. The immune system also has the capacity to detect and respond to aberrant cell death which is not strictly associated with pathogens. Immunogenic cell death (ICD) is a form of cell death that involves the release of endogenous DAMPs which are immunostimulatory [35]. These can include proteins (ex. heat shock proteins, HMGB1), DNA or other molecules such as ATP and uric acid which are hidden from the immune system in non-pathological conditions [35,36]. Both PAMPs and DAMPs (collectively be considered as ‘danger signals’) serve to activate the innate immune response and induce the maturation of professional antigen presenting cells (APCs) such as dendritic cells (DCs) [35,36]. Mature DCs migrate to secondary lymphoid organs and present antigen to the adaptive immune system in the context of costimulatory signals which induce the activation of an adaptive response [37]. OVs can provide both PAMPs and DAMPs to activate the innate immune system and prime an adaptive response. Owing to their microbial nature, most OVs are inherently immunogenic and will thus trigger PRRs [38,39,40]. For example TLR3 and 9 play an important role in immune activation in the context of oncolytic parvovirus infection [41]. Interestingly, the cell death induced by OVs has frequently been shown to be immunogenic and involve the release of a variety of DAMPs [35]. For example oncolytic VacV infection has been shown to induce the release of HMGB1 [42] while adenovirus treatment has been associated with the release of uric acid and concomitant activation of DCs which in turn leads to an adaptive antitumor response [43]. Such priming of an adaptive immune response via OV infection has been observed with a variety of other viruses including reovirus [44], VSV [45] and oncolytic measles virus [46]. Thus the resulting detection of danger signals can alter the inflammatory milieu of the tumor, transitioning from an immunosuppressive, non-inflamed status to an immunostimulatory, inflamed status [47] which has the potential to prime an adaptive response (Figure 1).

### 3.2. Provision of Antigens

OV infection also serves to enhance the repertoire of tumor-associated antigens (TAA) to which an immune response can be mounted. Signals derived from virally infected cells can promote cross-presentation of endocytosed antigens by DCs and thereby generate a CD8 T-cell response [48,49]. In a similar fashion infection of tumor cells by OVs has been demonstrated to result in the cross-priming of an immune response against TAAs [46,50]. Phagocytosis of mesothelioma cells infected with an oncolytic measles virus has been shown to induce maturation and cross presentation in DCs whereas pulsing of DCs with uninfected or UV-killed cells was unable to do so [46]. Furthermore, oncolysis has been shown to stimulate an antitumor immune response directed against neoantigens derived from mutated cancer proteins. In doing so, OV therapy was shown to synergize with checkpoint blockade therapy. In this context, full oncolysis was necessary as TLR agonist treatment with polyIC and CpG were both unable to induce an appreciable response when injected intratumorally [51].

### 3.3. Enhanced Recruitment

While activation is a crucial component of the OV-mediated immune response, the trafficking of additional immune cells to the tumor also represents a potent mechanism of oncolytic virus immunotherapy. Chemokines and cytokines are the immune system’s signals for cell recruitment to a site of infection. OVs are demonstrably capable of inducing the expression of these signals within the tumor as well as their receptors on relevant immune cells. VSV and VacV infection of tumors has been shown to result in the production of the pro-inflammatory chemokines CXCL1 and CXCL5 which mediate neutrophil recruitment and enhanced therapeutic outcome [52]. Gujar et al. demonstrated that reovirus infected prostate cancer cells produce a plethora of pro-inflammatory cytokines including IL-1α, IL-6 and GM-CSF which translated to increased lymphoid cell recruitment in vivo [53]. Similarly, NDV infection has been shown to enhance the number of myeloid, NK, NKT and conventional T cells within bilateral tumors, even when only one tumor harbours the replicating virus. Interestingly while this latter study implicates a role for general inflammation, superior infiltration of uninjected tumors was observed when tumors were of the same origin as opposed to heterologous tumors, indicating an antigen-dependent component [54].

Despite increasing evidence that much of the therapeutic benefit observed by OVs is the result of their orchestration of an antitumor immune response, when administered as monotherapies, OVs have yet to consistently demonstrate the desired clinical outcome. Though the existence of an OV-mediated immune stimulation is well documented, it appears that this alone is insufficient to produce reliable cures in the clinic. However, in understanding the strengths and weaknesses of the OV-induced immune response, one can begin to rationally combine these agents with a variety of other immunotherapies in order to play to the strengths and compensate for the weaknesses of either approach alone. This endeavour has met with early success across virtually all forms of immunotherapy and much of the most promising preclinical and clinical data using OVs comes from such strategies.

## 4. Engineering Oncolytic Viruses to Enhance Their Immunostimulatory Potential

Given that a key mechanism of OV therapy involves the induction of a cancer-targeted immune response but that this response is typically insufficient to mediate durable remission, a logical next step involves the genetic modification of OVs to enhance their immunostimulatory ability. To this end a plethora of cytokine-engineered OVs have been generated with the goal of further enhancing immune recruitment and activation beyond what is achieved by any OV naturally [9]. Cytokines themselves such as type I interferon and IL-2 have been extensively applied for the treatment of cancer in a variety of studies and as a part of standard of care therapies. While some effectiveness has been observed, these results are frequently associated with severe toxicities [55,56]. One conceptual advantage to OV-encoded cytokines as opposed to systemically applied cytokines is in localizing their effects to the tumor; potentially obviating much toxicity. Furthermore, infection of a tumor with a cytokine-producing virus provides a continued source of the cytokine in question and therefore restricts the need for continued administration. Ultimately the synergy observed between OVs and immunostimulatory cytokines provides strong rational for the continued application of cytokine-expressing OVs.

One of the most commonly used cytokines in OV backbones has been the granulocyte macrophage colony stimulating factor (GM-CSF) [9]. GM-CSF has been shown to induce highly effective antitumor immune responses [4,57] which has been attributed to improved antigen presentation through the recruitment and differentiation of professional APCs [58,59]. Indeed the first FDA approved OV, Talimogene laherparepvec (T-VEC) is an ICP34.5/ICP47 deleted HSV-1 virus which expresses GM-CSF [5]. T-VEC was shown in a phase II clinical trial to mediate a T-cell dependent immune response against injected tumors as well as uninfected metastases indicating a role in priming systemic antitumor immunity [60]. In a phase III clinical trial, T-VEC produced a drastically improved durable response rate of 16.3% as compared to 2.1% with GM-CSF alone. Additionally, overall survival was extended from 18.9 to 23.3 months [4]. Thus OVs do have the capacity to elicit therapeutically meaningful antitumor immunity in a clinical setting in certain instances. A variety of other OVs have been engineered to express GM-CSF and are at various stages of testing. Oncolytic VacV [61] and adenoviruses [62] expressing this cytokine have demonstrated induction of an immune response against injected and non-injected tumors in patients with metastatic disease. Cerullo et al. attributed these results to the activities of DCs as well as the stimulation of NK cells [62]. In a preclinical model of a measles virus expressing GM-CSF the authors highlighted the recruitment of neutrophils as an important component of the virus’ antitumor effects. It should be noted however, that this work was done in SCID mice therefore the effects of NK and DCs may overwhelm that of neutrophils in fully immunocompetent models [63]. Indeed, in another study T-cells were demonstrated to be critical to the lasting antitumor protection induced by a VSV-expressing GM-CSF [64].

A variety of other cytokines have been engineered into OV backbones in efforts to increase immune activation. IL-2 has been shown to enhance a T-cell response in animal models of cancer but its’ systemic application has come with a plethora of toxicities [65]. In a screen in which a variety of immunostimulatory cytokines were engineered into the NDV backbone, NDV expressing IL-2 was able to produce remissions in the greatest number of treated mice. The authors demonstrated that IL-2 expression was restricted to the tumor and locally increased T-cell infiltration [66].

Another immune activating group of cytokines are the interferons (IFNs). Though accompanied by significant clinical side effects, systemic IFN-α administration has been used directly as a treatment for a variety of malignancies. In addition to direct antiproliferative effects on tumors, it also has immune activating properties in terms of MHC I upregulation and direct effects on cells of the immune system such as macrophages and NK cells [67,68]. In a hamster model of pancreatic cancer, an oncolytic adenovirus expressing IFN-α displayed significantly improved therapeutic efficacy when compared to controls expressing luciferase, although the direct antitumor effects rather than immunostimulatory activities of this cytokine were highlighted [69]. In contrast the incorporation of IFN-β into a VSV backbone provided therapeutic benefit in a fashion shown to be T-cell dependent. However, this was attributed to a generalized increase in T-cell activation as opposed to an antigen-specific activity [70]. In addition to type I IFNs, the expression of the type II IFN (IFN-γ) has demonstrated clear therapeutic benefit in a variety of models. For example, an IFN-γ expressing VSV was demonstrated to better activate DCs in a 4T1 mammary cancer mouse model which translated to an improved therapeutic benefit in a T-cell dependant fashion [71]. Authors who investigated the efficacy of an oncolytic adenovirus encoding IFN-γ attributed the therapeutic benefit of such an agent to oncolysis as well as the antiangiogenic and immune stimulatory effects. Indeed, improved T-cell infiltration was clearly observed in liver cancer models [72].

A plethora of other immunostimulatory cytokines have been engineered into various OV backbones, a comprehensive list of which can be found in [9]. Another strategy has been the engineering of immune-recruiting chemokines into viral genomes. For example, CCL5 when expressed from an oncolytic VacV, was able to increase tumor infiltration of DCs and CD4+ but not CD8+ T-cells. In the context of a DC vaccination primed response however, VacV expressing CCL5 did significantly augment the tumor T-cell recruitment. This demonstrates the variability in immune response produced depending on the manner of use of the chemokine-expressing OV [73]. An oncolytic adenovirus expressing the same chemokine was shown to mediate therapeutic benefit which was associated with improved DC, macrophage, NK and CD8+ T-cell recruitment [74]. Similarly, a combination therapy using adenovirus vectors engineered to express CCL3 and FLT3L in concert with an oncolytic conditionally replicating adenovirus displayed a superior therapeutic efficacy through improved DC and T-cell recruitment/activation despite inducing a more potent antiviral response [75]. While the incorporation of cytokines and chemokines into OV backbones has clearly demonstrated therapeutic benefits both in clinical and preclinical trials, the pleiotropic effects of these molecules can complicate their use in such a context [76]. More direct approaches have thus been taken such as encoding bispecific T-cell engagers (BiTEs) into oncolytic viral genomes. BiTEs are composed of a single chain variable fragment (scFv) targeted to a selected antigen linked to another scFv targeted to the CD3 chain of the T-cell receptor. Thus they can mediate T-cell activation in response to a defined target through the artificial linking of the TCR to an epitope not derived from its’ cognate antigen [77]. For example, a VacV engineered to express a BiTE targeting the EphA2 TAA was able to promote a T-cell response against cells expressing this antigen and thus mediate the killing of cancer cells that were not infected by the virus [78].

As demonstrated above, strategies aimed at increasing the immunostimulatory potential of OVs clearly produce therapeutic benefits but have yet to achieve the sought after durable remissions. Therefore, evidence for the augmented effect of combining these agents with other therapies such as cancer vaccines [9] is encouraging as it unveils a plethora of new directions through which the benefit of OVs and their immune-engineered counterparts can be further realized.

## 5. Oncolytic Viruses in Cancer Vaccination

The description of TAAs as potentially cancer-specific targets [79] spawned a plethora of approaches designed at eliciting an immune response against these epitopes. Based on the enormous success of classical vaccination strategies, one such notion was the creation of ‘cancer vaccines’ which encompass DNA, RNA, peptide, cell and vector mediated strategies (reviewed elsewhere [80]). However much of the clinical data from cancer vaccine based approaches has been disappointing [81]. The choice of targets, adjuvants, delivery methods and the ability to overcome tumor-mediated immunosuppression have emerged as critical elements moving forward [82].

From a conceptual standpoint, OVs have the potential to address several of these criteria and thus synergize with a variety of these vaccination approaches. The anti-cancer immune response elicited by OVs upon infection and induction of immunogenic cell death within the tumor has been termed in situ vaccination [9]. In this way OVs themselves can be thought of as cancer vaccines. For example, Moehler et al. demonstrated that infection with oncolytic parvovirus induces DC maturation and subsequent cross-presentation of tumor cell antigens in vitro [83] which would be a pre-requisite for induction of an antitumor immune response. The ensuing adaptive immune response has been demonstrated for a variety of OVs [26,27,28] and can be thought of as a ‘vaccination’ effect that, while important to OV action, is insufficient to mediate cures in most cases of advanced disease. Indeed, Toda et al. noted the establishment of a tumor-specific immune response following treatment of CT26 or M3 tumors with oncolytic HSV-1. They described this phenomenon as in situ vaccination as it depended on infection of tumors with the OV [84].

The natural vaccinating propensity of OVs can also be enhanced through further manipulation of the viruses. For example, Lemay et al. demonstrated that infecting an irradiated cancer cell vaccine with oncolytic VSV prior to its administration led to an improved therapeutic outcome when compared to the cell vaccine alone; an effect which the authors attributed to the adjuvant effects of the virus. Indeed, vaccine-mediated protection was enhanced when the virus was engineered to express GM-CSF and was mediated through improved activation of DCs and a concomitant increase in T and NK cell responses [85]. In another strategy, VSV was used as a delivery platform for an RNA vaccine in mouse models of prostate cancer. In this study, cDNA from human prostate was encoded into the VSV genome and the viruses were subsequently used to treat murine TC2 tumors producing cure rates of 80%. Although some mice did relapse with escape variants, these could be treated using viruses engineered with cDNA from the escape mutants. Crucially, the initial therapy was more effective when using cDNA derived from human as opposed to mouse prostate [86]. Thus the general vaccinating ability of OVs can also be enhanced through xenoimmunization.

One strategy to direct the vaccinating effect of OVs has been to generate recombinant OVs which encode a tumor-associated antigen in the viral genome, termed oncolytic virus vaccine (OVV). This provides the induced immune response with a defined target as opposed to relying on the release of antigens upon tumor cell lysis. For example, expression of HY (a TAA expressed in the MB49 murine urothelial carcinoma cell line) by VacV has been used in conjunction with a VacV expressing GM-CSF to induce a T-cell response against this target antigen [87]. Despite the conceptual elegance of such a strategy, the desired antitumor immune response is frequently eclipsed by a more potent antiviral response when the targeted TAA is self-derived while the viral antigen is completely foreign [88].

**Figure 2:**
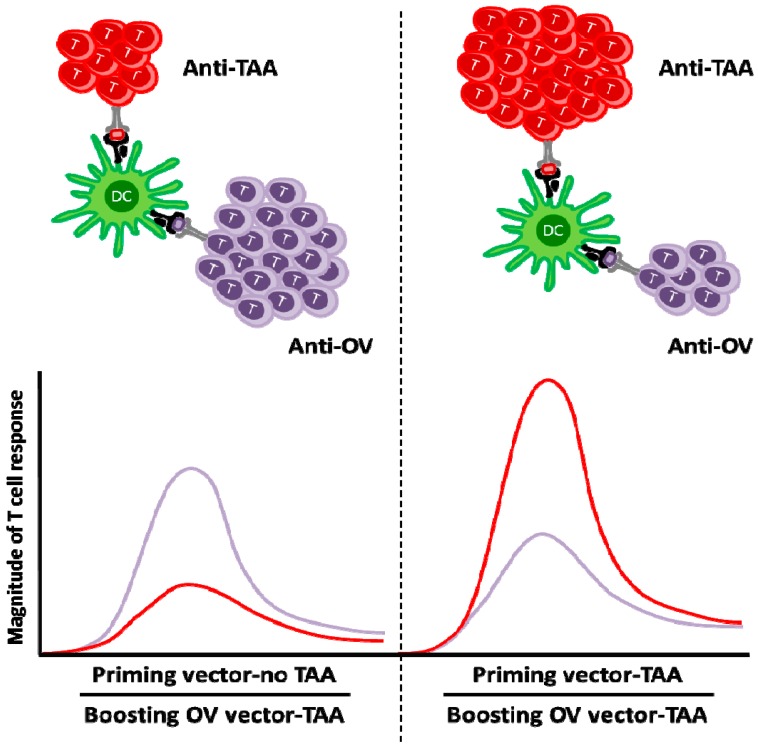
Left side: Enhanced anti-tumor response when using OV to boost a TAA prime. When boosting an anti-TAA response with an OV, two distinct viral vectors encoding a common tumor-associated antigen are employed. Priming with an empty adenoviral vector (Priming vector-no TAA) and boosting with a rhabdovirus expressing a TAA (Boosting OV vector-TAA) induces an anti-TAA response which is overshadowed by the anti-OV response; Right side: Priming with an adenoviral vector expressing a TAA (Priming vector-TAA) and boosting with a rhabdovirus expressing the same TAA (Boosting OV vector-TAA) induces a dramatically enhanced anti-TAA response and a reduced anti-OV response.

In order to circumvent the antiviral immune response and achieve a robust antitumor response in the context of OV vaccination, a heterologous prime-boost strategy has been taken [89]. In this approach, a primary response to a defined tumor antigen is established using a priming vector expressing this antigen. Subsequently, an antigenically distinct OV encoding the same tumor antigen is administered as a boosting vector. In such a context the secondary immune response against the tumor antigen eclipses the primary immune response against the OV (Figure 2). This was demonstrated in a murine B16 melanoma model that expresses the dopachrome tautomerase (DCT), a melanocyte-differentiation antigen. An adenoviral vector expressing human DCT (hDCT) induced a substantial level of antitumor immune response that was effectively boosted by an oncolytic VSV expressing the same antigen. This combined approach provided a greater therapeutic benefit than either agent alone through the induction of a more potent CD8+ T-cell response. It was also shown that the magnitude of the anti-VSV response was actually reduced in the prime-boost regimen, leading to enhancements in both efficacy and safety [89]. Remarkably the magnitude of the anti-DCT T-cell response was greater in tumor-bearing animals than in tumor-free animals, which was accompanied by significantly greater numbers of antigen-specific tumor-infiltrating T-cells; reinforcing the advantage of using a replicating OV as a boosting vector for treating established tumors. Interestingly, oncolytic VSV also increases production of TNFα, IFNγ and granzyme B by antigen-specific T-cells, suggesting that oncolytic vaccines may provide additional benefits to enhance T-cell quality [90].

**Figure 3:**
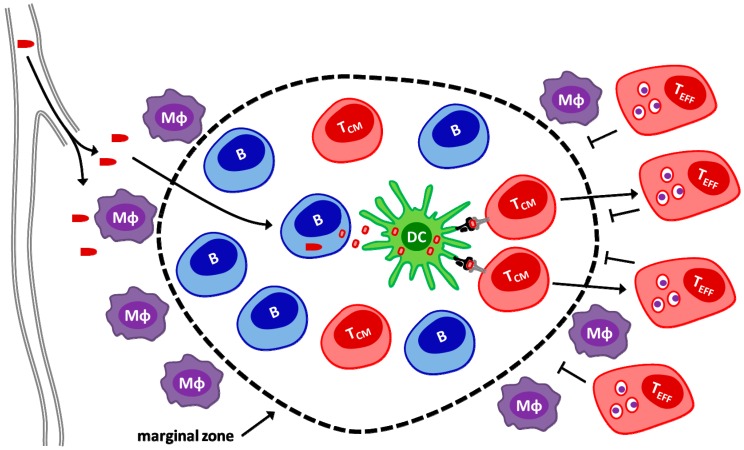
Rhabdovirus OVV boosting of T_CM_ in the splenic follicle and the marginal zone as an anatomical barrier preventing T_EFF_ recirculating back to the follicle. TAA-specific T_CM_ cells induced by the priming vector reside in the splenic follicle which is maintained as an immunopriviliged site by the surrounding marginal zone and its resident marginal zone macrophages (MΦ). When administered by intravenous injection, VSV traffics to the splenic follicle and infects follicular B cells. Infected B cells produce and release TAA encoded by the rhabdovirus which is taken up by neighbouring DCs and presented to T_CM_ cells. Stimulated T_CM_ cells are converted into T_EFF_ and are excluded from the follicle by the marginal zone so that they cannot eliminate TAA carrying DCs.

hDCT-expressing Maraba MG1 was also demonstrated to be a strong boosting vector, producing higher levels of activated CD8+ T-cells than did VSV boosting. Interestingly, Maraba MG1-hDCT had improved boost at shorter intervals and required intravenous administration to provide its’ full effect [91]. This was surprising as one might expect migratory antigen presenting cells to be killed prior to trafficking to secondary lymphoid organs and performing their antigen presenting functions if a primary response with effector T cells (T_EFF_) targeting hDCT was still underway [92]. Subsequent mechanistic studies by Bridle et al. [93] have shown that I.V. injection of VSV or Maraba MG1 in mice leads to direct infection of follicular B-cells in the spleen (Figure 3). These infected B cells serve as a source for antigen production which is subsequently captured by neighbouring CD11c^+^ DCs and presented to central memory T cells (T_CM_) that are also located within B cell follicles. Since effector T-cells cannot traffic through follicular regions (prevented by marginal zone), prolonged antigen production and presentation by follicular APCs is secured [93]. This unique mechanism based on rhabdoviral tropism for splenic follicular B-cells allows the shortening of the interval between prime and boost which could have relevance in the application of such a strategy with regard to highly aggressive cancers in the clinic. This prime-boost strategy is currently being explored in a clinical trial using adenoviral prime and Maraba MG1 boost strategy targeting the MAGE-A3 antigen [94].

## 6. Adoptive Cell Transfer and Oncolytic Viruses: Orchestrating the Attack

The use of heterologous prime-boost protocols has clearly demonstrated the potential of oncolytic vaccines to recruit and expand a pre-existing (primed) anti-tumor response [89,90]. Priming of the immune response however need not be restricted to the use of a viral vector. For example, Chuang et al. treated OVA-expressing tumors with an oncolytic VacV encoding the same antigen after priming with a DNA vaccine [95]. Given that other boosting strategies have successfully utilized pre-existing antitumor immunity as a platform for boosting [96], it has even been suggested that in some cases the tumor alone may serve as sufficient priming for boosting by oncolytic vaccines [97]. While this represents an exciting prospect it is likely that endogenous responses, if present, would not be of sufficient magnitude or quality to mediate appreciable therapeutic benefit in many cases.

One way to ensure the presence of a robust pre-existing antitumor T-cell response would be to directly transfer primed T-cells into the patient. Adoptive cell therapy (ACT) as it is known, involves the administration of ex vivo cultured cancer targeted T-cells to mediate cancer killing. These T-cells can be inherently TAA-specific lymphocytes isolated from a patient’s tumor or enriched from peripheral blood monocytes (PBMCs). They can also be cells that have been artificially reprogramed for cancer targeting through various recombinant DNA technologies [98,99]. Such approaches have recently demonstrated considerable success in the targeting of hematological malignancies [100,101] with the results in solid tumors being less impressive [102,103]. One proposed reason for the decreased efficacy of ACT in solid tumors involves impediments to the efficient trafficking of adoptively transferred cells to the tumor [104]. In this respect, the inflammatory environment established upon OV infection could serve to facilitate the recruitment of these cells. For example, Fu et al. showed that infection of an OVA-expressing pancreatic cancer model with an oncolytic HSV-2 enhanced both recruitment and persistence of adoptively transferred OVA-specific T cells and contributed to an improved therapeutic outcome in a manner that was attributed to local upregulation of inflammatory chemokines such as CXCL9 and CXCL10 within the tumor [105].

**Figure 4:**
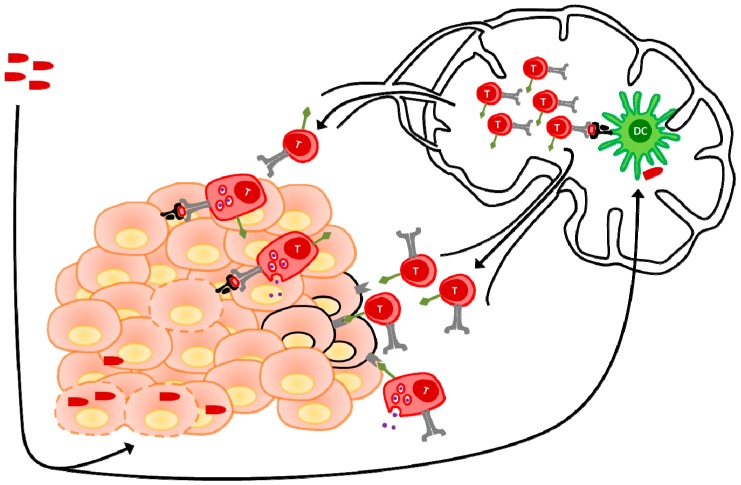
Combination of dual-specific ACT and OVV therapy. T cells with native TCR specificity for a TAA are engineered to express a recombinant TCR or CAR in order to generate dual specific T cells for ACT. Serial injection of dual specific T cells and an OVV serves to activate T cells through TCR stimulation and recruit them to the tumor where they can detect TAA positive cells and attack the tumor through either TCR or CAR binding of its target. In this way, tumors with heterogeneous TAA expression can be effectively targeted and destroyed with one combination therapy.

Another major difficulty faced in the effective clinical use of ACT has been the poor persistence of cells following transfer [106]. While initial work in the field involved the use of highly activated and cytolytic effector cells, more recent approaches have focused on culturing cells for a less differentiated memory phenotype, which has since been shown to mediate superior clinical outcomes [107]. In such a context, OVVs seem ideally poised to synergize with ACT since the cells being transferred could be viewed as primed CTLs specific for the antigen encoded by the OVV and awaiting boosting. For example, CD62L-expressing central memory T-cells (T_CM_) will traffic to secondary lymphoid organs upon adoptive transfer but could theoretically be driven to proliferate and subsequently recruited to the tumor through boosting with an OVV, just as previously described in the prime-boost therapy platform [93]. Because the OVV can drive expansion in vivo this approach has another conceptual advantage over the transfer of highly activated effectors in that transfer of smaller numbers of cells should be possible and the need for continued administration of IL-2 to the patient following transfer is obviated. Proof of principle for the use of viruses in expanding tumor-specific T-cells has come from studies which generated dual specific T-cells [108,109]. In these approaches T-cells specific for viruses which have established latent infections in a large proportion of the human population [108] or viruses with which experimental mice were immunized [109] were isolated and transduced with a TCR [108] or CAR [109] specific for a TAA. In both cases, presentation of viral antigen to the T-cells drove their expansion while tumor-cell killing could be mediated via the recombinant TCR or CAR [108,109] (Figure 4) These studies demonstrate the potential of utilizing viruses to drive tumor-reactive T-cell expansion. The use of an OVV in this context offers the additional possibility of direct infection of the tumor which would be advantageous in recruitment of T-cells [109]. The use of lymph-node trafficking memory T-cells in such approaches would limit the possibility of feedback inhibition in which circulating effectors kill migratory DCs before they can reach secondary lymphoid organs to activate a memory response [110]. Thus such a strategy would circumvent any instance in which excessive primary responses resulted in detriment to the boost.

While OVs clearly have the capacity to orchestrate and enhance the antitumor immune response elicited by adoptively transferred cells, ACT may also provide a means for enhancing the efficacy of OV therapy. Achieving an optimal delivery method has presented a recurrent challenge in virotherapy with systemically administered viruses falling prey to an array of neutralizing factors prompting the investigation of strategies such as cell carriers designed to cloak and deliver OVs to the tumor [111]. As an extension of this concept, Qiao et al. used antigen specific adoptively transferred cells as OV carriers to combine the effects of OV therapy and ACT. This approach proved highly synergistic with adoptively transferred cells effectively delivering virus to the tumor while acquiring enhanced activation and in vivo function, resulting in a more potent endogenous antitumor response than either therapy alone [112]. Additionally, VanSeggelen et al. showed that OV loading can be efficiently combined with CAR-engineered T-cells and that such an approach can be used to target cancer cells to which are resistant to either of the therapies alone [113].

## 7. Taking off the Breaks with Checkpoint Inhibitors

Currently, the most clinically advanced form of cancer immunotherapy is checkpoint blockade with two agents in this category having FDA approval for use in advanced melanoma and undergoing trials for a variety of other malignancies [114]. Immune checkpoints are a means by which the body, under normal circumstances, uses to provide inhibitory signals to CTLs and thus ensure self-tolerance and limit immune pathology. These signals however are commonly upregulated within the tumor microenvironment allowing the cancer to dampen local immune response and limit antitumor immunity [115].

Cytotoxic T-lymphocyte-associated protein 4 (CTLA-4) and programmed death ligand 1 (PDL-1) represent T-cell expressed checkpoint receptors extensively studied for their role in tumor immunosuppression [115,116,117]. CTLA-4 binds the same B7 ligands as the CD28 costimulatory receptor but more strongly and without providing a costimulatory signal therefore impairing the signal 2 necessary for T-cell activation [115,117]. Programmed cell death-1 (PD1) displays a wider array of expression on immune cells than CTLA-4 and can impede both activation and effector function on T-cells [115,116,117]. Tumors frequently upregulate the PD1 ligands PD-L1 and PD-L2 in order to dampen T-cell responses [115,117].

Immune checkpoint blockade is a strategy to target and block these immunoregulatory mechanisms with the use of antibodies in order to ‘release the brake’ on the immune response to TAAs [118]. Examples of immune checkpoint blockade include ipilimumab (a CTLA-4 inhibitor) and nivolumab (a PD-1 inhibitor), both of which have gained FDA approval for treatment of metastatic melanoma [116]. Despite clinical excitement for the use of the checkpoint blockade, this treatment modality does not generate an antitumor immune response so much as remove impediments on an already established immune response. For example, van Elsas et al. demonstrated that in the context of a poorly immunogenic B16F10 melanoma model, effective anti-CTLA-4 therapy required the administration of a GM-CSF expressing cell vaccine alongside checkpoint blockade to achieve appreciable impact [119]. As such it is not surprising that ipilimumab is more effective in patients with higher expression of immune related genes [120]. Similar findings have been reported for strategies targeting PD-1 [121]. Therefore there exists clear potential for synergy between OVs and checkpoint blockade. Broadly speaking OVs offer the possibility of priming, boosting and effector recruitment in the context of an antitumor response while the checkpoint blockade is demonstrably effective in enhancing the potency of such a response through the removal of inhibitory factors.

Several proof of principle examples of the potency of a combined OV-checkpoint blockade therapy are now emerging. For example, Zamarin et al. demonstrated in mouse models that a combination of oncolytic NDV and anti-CTLA4 could cure the majority of animals while NDV alone was effective in only 10% of mice. Interestingly, this strategy could induce an immune response against both virally injected and uninfected tumors, though this effect was impeded in tumors that were antigenically distinct. These effects were CD8+ T-cell, NK cell and type I IFN-dependent but independent of oncolysis [54]. Rojas et al. provided additional insight into the design of effective combinations of OV and checkpoint therapy in the context of VacV and anti-CTLA4 therapy. In this study, timing of immune checkpoint blockade administration after OV delivery proved to be an essential part of the overall therapeutic efficacy. The authors demonstrated improved therapeutic efficacy when the checkpoint blockade is administered four days following OV treatment. They attributed this finding to a reduction in the potency of the antiviral response when the virus is not given in conjunction with the checkpoint inhibitor. In such a regimen, combination therapy increased both the overall magnitude of tumor-specific T-cells as well as those infiltrating the tumor [122]. In an effort to alleviate some of the off-target toxicities associated with enhanced immune activation in the checkpoint blockade, Engeland et al. elected to directly encode antibodies targeting CTLA4 and PD-1 into the backbone of an oncolytic Measles virus. Though both viruses were capable of reducing tumor size, the greater survival benefit was seen with measles virus encoding a PD-L1 antibody through an enhanced CD8+ T-cell response. Treatment with the viruses encoding antibodies was comparable to administration of virus and antibody separately but both combinations were superior to either therapy administers as a single agent [123].

Thus the combination of oncolytic viruses and checkpoint blockades has demonstrated significant promise in enhancing the efficacy of either therapy alone. However, such an approach does not exclude the combination with other forms of immunotherapy. For example, alleviation of checkpoint induced immune suppression could prove beneficial in the context of the prime boost or ACT + OVV strategies previously discussed. While these combinations could bring a new level of potency to these approaches, such endeavours should be undertaken with care as the alleviation of immune checkpoints through checkpoint blockade is largely non-specific [114] in its current use and could therefore, enhance potential autoimmune effects in these therapies.

## 8. Future Perspectives

As it becomes increasingly clear that a large portion of the efficacy of OV therapy stems from its modulation of an antitumor immune response, it is likely that efforts in the field will increasingly focus on harnessing this potential. While this can and should be undertaken in a variety of different ways, including further engineering of these vectors, recent experience clearly illustrates the potential of such approaches to synergize with other immunotherapeutic strategies. Given the broad spectrum of available OVs and the ease with which these agents can be further engineered for specific needs, they provide unparalleled potential and versatility as tools. As combinational agents, they offer the potential to play to the strengths and compensate for the weaknesses of other immunotherapeutic strategies, illustrating true synergy. Viewed in such a context, OVs can represent a foundation on which diverse therapeutic strategies can be rationally built in order to address the highly heterogeneous disease that is cancer.
